# Suppression of lung adenocarcinoma migration through organelle alkalization by human lactoferrin – albumin fusion

**DOI:** 10.1002/2211-5463.70237

**Published:** 2026-03-24

**Authors:** Hana Nopia, Masahiro Kimura, Daisuke Kurimoto, Atsushi Sato

**Affiliations:** ^1^ School of Bioscience and Biotechnology Tokyo University of Technology Japan

**Keywords:** Cancer migration, epithelial–mesenchymal transition (EMT), human lactoferrin–albumin fusion (hLF‐HSA), matrix metalloproteinase 1 (MMP1), Na^+^/H^+^ exchanger 7 (NHE7), organelle pH

## Abstract

The fusion of human serum albumin with human lactoferrin (hLF‐HSA) exerts strong anti‐migratory effects in human lung adenocarcinoma cells through matrix metalloproteinase 1 (MMP1) downregulation. We demonstrate that hLF‐HSA disrupts organelle pH homeostasis via Na^+^/H^+^ exchanger 7 (NHE7) upregulation, inducing organelle alkalization in the Golgi apparatus. This organelle dysfunction alters the Golgi‐mediated secretome, leading to MMP1 downregulation and suppression of cell migration. hLF‐HSA‐induced MMP1 downregulation also reverses epithelial‐mesenchymal transition (EMT), further suppressing migration. Additionally, hLF‐HSA‐driven activation of caveolae‐mediated endocytosis (CavME) signaling downregulated MMP1 expression without NHE7 upregulation. These findings highlight that hLF‐HSA–mediated disruption of organelle pH regulation and CavME activation represents a potential strategy to suppress cancer cell migration.

**Impact statement**

This work reveals a previously unrecognized link between organelle pH dysregulation and cancer cell migration. By identifying hLF‐HSA as a dual modulator of Golgi function and endocytic signaling, it provides a mechanistic basis for developing novel anti‐metastatic strategies targeting intracellular trafficking rather than cytotoxicity.

## Graphical abstract

This paper reveals how human lactoferrin–albumin fusion (hLF‐HSA) potently suppresses lung adenocarcinoma cell migration. hLF‐HSA upregulates NHE7, leading to Golgi alkalization, disruption of the Golgi secretome, downregulation of MMP1, and reversal of EMT. These findings suggest a novel Golgi‐targeting strategy to suppress cancer cell migration.

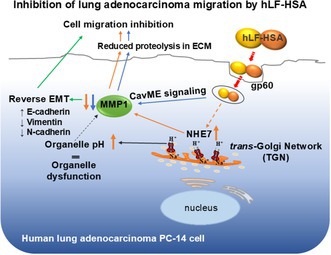

**Abbreviations**
(Cav1): caveolin‐1
(CavME): caveolae‐mediated endocytosis
(ECM): extracellular matrix
(EMT): Epithelial–mesenchymal transition
(hLF‐HSA): Human lactoferrin–albumin fusion
(MMP1): Matrix metalloproteinase 1
(NHE7): Na^+^/H^+^ exchanger 7

Cancer arises from the transformation of normal cells into abnormal cells through a multistage process, eventually forming malignant tumors that can metastasize to other organs [1]. Cancer encompasses a large group of diseases that can occur in diverse areas of the body and is a leading cause of death worldwide, primarily because of the widespread metastasis of cancer cells [2]. Therefore, the identification of novel strategies to inhibit cancer metastasis could pave the way for more effective treatments [3].

The Golgi apparatus is an important cellular organelle that plays a crucial role in protein processing and trafficking by modifying and sorting proteins based on their specific cellular destinations [4]. It ensures that proteins are properly processed and secreted, affecting various cellular processes including signaling, metabolism, and interactions with the extracellular environment [5]. The Golgi apparatus is responsible for secreting matrix metalloproteinases (MMPs) that contribute to the formation of the extracellular matrix (ECM). MMPs are the primary enzymes involved in ECM remodeling, degrading components such as collagen to clear a path in the ECM and allowing cells to move into adjacent tissues and beyond [6]. ECM remodeling provides structural and biochemical conditions that facilitate cancer cell migration, which is a critical step in cancer metastasis [7]. Therefore, the dysregulation of the Golgi apparatus may be a promising target for the development of anti‐migratory agents [8]. The disruption of pH homeostasis could be a strategy because the mildly acidic environment is tightly regulated [9, 10]. However, a major concern is that the Golgi apparatus also functions in normal cells [5]. Notably, there are currently no anticancer drugs specifically approved to target the Golgi apparatus [11].

Na^+^/H^+^ exchanger 7 (NHE7) is an isoform of organelle membrane sodium‐hydrogen exchangers (NHEs) predominantly localized in the *trans*‐Golgi network (TGN), where it regulates the resting pH of the TGN [12]. NHE7 typically facilitates the exchange of sodium ions with protons across the membranes. It has been proposed to function as a proton‐leak pathway in lung adenocarcinoma [10] and as a proton‐loading pathway in pancreatic adenocarcinoma [9, 10]. However, the contrasting roles of NHE7 in different cancer cell types remain poorly understood [13]. Elevated NHE7 expression is frequently observed in tumors and is associated with malignant progression [9, 14, 15, 16]. The therapeutic benefits of pharmacological blockade or genetic inactivation of NHE7 have been shown in various cancer models [9, 14, 15, 16], indicating that selective NHE7 inhibition could serve as a potential therapeutic strategy.

Lactoferrin (LF), a protein involved in innate immunity, has been reported to exert anti‐proliferative, anti‐migratory, and anti‐metastatic effects on cancer cells [17, 18]. In a recent study, we successfully produced a human serum albumin (HSA)‐fused human lactoferrin (hLF) fusion protein (hLF‐HSA) using Chinese hamster ovary (CHO) cells, which enhanced cellular entry into cancer cells in a caveolae‐dependent manner [19]. hLF‐HSA has been shown to exhibit stronger anti‐proliferative activity against human lung adenocarcinoma PC‐9 [10] and PC‐14 [19] cells than hLF, owing to organelle alkalization induced by hLF‐HSA‐elicited NHE7 upregulation. Notably, they exhibit selective growth‐inhibitory effects on cancer cells without affecting normal cells [20]. Therefore, hLF‐HSA is a potential Golgi‐targeting anticancer protein [10].

Overall, hLF‐HSA showed robust anti‐migratory effects in PC‐14 cells by suppressing MMP1 expression [21]. In the present study, we investigated whether the inhibitory effects of hLF‐HSA on PC‐14 cell migration were attributable to NHE7 upregulation‐induced organelle alkalization, despite previous reports linking elevated NHE7 expression with cancer progression [9, 14, 15, 16]. Furthermore, we discuss the involvement of hLF‐HSA‐induced caveolae‐mediated endocytic signaling in its anti‐migratory effects.

## Materials and methods

### Reagents and chemicals

The holo form of iron‐saturated recombinant hLF (iron content, 1588 ng/mg protein) derived from *Aspergillus niger* (> 95% purity) was prepared as previously described [21]. HSA (017‐10 504, > 95% purity) was purchased from the Fujifilm Wako Pure Chemical Corporation (Osaka, Japan). hLF‐HSA (holo form; iron content: 1302 ng/mg hLF equivalent) was produced in CHO cells as described previously [20]. Okadaic acid was purchased from Cayman Chemical Company (Ann Arbor, MI, USA). Lipofectamine RNAiMAX was purchased from Thermo Fisher Scientific (Waltham, MA, USA). Cell culture reagents were purchased from Nacalai Tesque, Inc. (Kyoto, Japan), unless otherwise specified.

### Cell culture

The human nonsmall‐cell lung carcinoma cell line PC‐14 (RRID:CVCL_1640) [22], registered in the ExPASy Cellosaurus database, was used in this study. PC‐14 cells were obtained from Immuno‐Biological Laboratories Co., Ltd. (Gunma, Japan), which provides authenticated and mycoplasma‐free cells. Upon receipt, this cell line was expanded once, aliquoted, and cryopreserved to establish a working cell bank. For all experiments, cells were thawed from this bank and used for a limited number of passages to minimize the risk of cross‐contamination and mycoplasma contamination. PC‐14 cells were cultured in Roswell Park Memorial Institute (RPMI) 1640 medium supplemented with 10% (v/v) fetal bovine serum (FBS) and incubated at 37 °C in a 5% CO_2_ atmosphere.

### Small interfering RNA (siRNA)‐mediated knockdown of MMP1, NHE7, and Cav1

siRNAs specific to MMP1, NHE7, and caveolin‐1 (Cav1) were used to knockdown their expression (Table 1). Lipofectamine RNAiMAX was used for transfection, according to the manufacturer's instructions. A negative siRNA control, as well as siMMP1‐, siNHE7‐, and siCav1‐transfected cells were incubated in RPMI‐1640 medium supplemented with 10% FBS for 48 h. Silenced cells were used for further experiments, including measurement of organelle pH, western blotting, and Boyden chamber assays.

**Table 1:** siRNA sequences.

siRNA	Sequences
siRNA control	siLuc sense 5′‐CGU ACG CGG AAU ACU UCG Att‐3′
	siLuc antisense 5′‐UCG AAG UAU UCC GCG UAC Gtt‐3′
siCav1	Sense 5′‐UUU CCC AAC AGC UUC AAA GAG UGG G‐3′
	Antisense 5′‐CCC ACU CUU UGA AGC UGU UGG GAA A‐3′
siNHE7‐5S	Sense 5′‐CGA AGU CUG CUU GAC UGC AAC CUC Att‐3′
	Antisense 5′‐UGA GGU UGC AGU CAA GCA GAC UUC Gtt‐3′
siMMP1S	Sense 5′‐GCA AAU GCA GGA AUU CUU Utt‐3′
	Antisense 5′‐AAA GAA UUC CUG CAU UUG C‐3′

### Measurements of intracellular organelle pH

Intracellular organelle pH was measured using the pH‐sensitive fluorescent probe LysoSensor Green DND‐189 (DND‐189; pKa, 5.2; working range, 4.5–6.0; Thermo Fisher Scientific, USA), as previously described [10]. PC‐14 cells transfected with the siRNA control and/or siNHE7‐ were seeded at 1 × 10^4^ cells/well in a 96‐well glass‐bottom plate in RPMI‐1640 medium supplemented with 10% FBS and cultured at 37 °C in a 5% CO₂ incubator overnight. The medium was then replaced with serum‐free RPMI‐1640 containing 5 μM hLF‐HSA and incubated for 4 h, after which the medium was replaced again with serum‐free RPMI‐1640 containing 1 μM DND‐189 and incubated for an additional 30 min. Green fluorescence in living cells was captured using confocal laser scanning microscopy (CLSM) FV3000 (Olympus, Tokyo, Japan). The imagej software (National Institutes of Health, Bethesda, MD, USA) was used to analyze the fluorescence intensity in each intracellular area.

### Boyden chamber assay

A Boyden chamber assay was performed using polycarbonate membranes with 8‐μm pores (Cat. no. 3422; Corning, Corning, NY, USA), as described previously [21]. Briefly, PC‐14 cells (2 × 10^5^) suspended in 400 μL serum‐free RPMI‐1640 with or without 5 μm hLF‐HSA were seeded into the upper chambers of a 24‐well plate. The lower chambers were filled with 750 μL RPMI‐1640 supplemented with 10% FBS. siRNA‐transfected cells were used as needed. After 48 h, nonmigrating cells on the upper surface were removed, and migrating cells on the undersurface were fixed with ice‐cold methanol, stained with 0.2% crystal violet, and washed with PBS. Migrated cells were photographed, and the stained area was quantified in five random fields using ImageJ.

### Western blotting

Western blotting was performed as previously described [21]. The specific antibodies used in this study are listed in Table 2.

**Table 2:** Antibodies.

Antibodies	Manufacturer and catalog number	Working dilution	Buffer
Anti‐MMP1 rabbit antibody	10371‐2‐AP, Proteintech, Rosemont, IL, USA	1 : 1000	5% skim milk in TBST
Anti‐Caveolin1 rabbit antibody	3238S, Cell Signaling Technology, Dancers, MA, USA	1 : 1000	1% BSA in TBST
Anti‐NHE7 rabbit antibody	ab272649, Abcam, Cambridge, MA, USA	1 : 2000	5% skim milk in TBST
Anti E‐cadherin rabbit antibody	24E10, Cell Signaling Technology, Dancers, MA, USA	1 : 1000	1% BSA in TBST
Anti‐vimentin rabbit antibody	D21H3, Cell Signaling Technology, Dancers, MA, USA	1 : 1000	1% BSA in TBST
Anti‐N‐cadherin rabbit antibody	GTX127345, Genetex, CA, USA	1 : 5000	5% BSA in TBST
Anti‐hLF rabbit antibody	A80‐144A, Bethyl Laboratories Inc, Montgomery, TX, USA	1 : 5000	1% BSA in TBST
Biotinylated anti‐HSA antibody	A114BN, American Qualex, San Clemente, California, USA	1 : 5000	1% skim milk in TBST
Anti‐rabbit IgG (H + L) horseradish peroxidase (HRP)	65‐6120, Promega corporation, Madison, WI, USA	1 : 10000	2% skim milk in TBST
Anti‐mouse IgG (H + L) horseradish peroxidase (HRP) conjugated antibody	W4021, Promega Corporation, Madison, WI, USA	1 : 10000	2% skim milk in TBST

### Construction of an expression vector for hLF, HSA, or hLF‐HSA

hLF expression vector: Full‐length hLF cDNA was excised from pBSIILfAL [23] using *Xho*I and *Not*I restriction enzymes. *Xho*I and *Not*I fragments were inserted into the corresponding sites of the pCI‐neo mammalian expression vector (Promega, Madison, WI, USA) to construct pCI‐neo hLF expression vector.

HSA expression vector creation: An *EcoR*I‐*Not*I fragment isolated from pOptiVEC/HSA‐hLF [20] was ligated into the corresponding sites of pCI‐neo to construct pCI‐neo HSA‐hLF. The HSA cDNA fragment was amplified using polymerase chain reaction (PCR) with pCI‐neo‐HSA‐hLF as a template, along with the following primers: HSA (1091‐1110), 5′‐ATTACTCTGTCGTGCTGCTG‐3′ and HSATAANotI, 5′‐CCGCGGCCGCTTATAAGCCTAAGGCAGCTTGAC‐3′. An *Xba*I‐ and *Not*I‐digested fragment isolated from the PCR products was inserted into the corresponding sites of pCI‐neo HSA‐hLF to generate the HSA expression vector, pCI‐neo HSA.

hLF‐HSA expression vector: hLF‐HSA cDNA from pOptiVEC/hLF‐HSA was digested [20] using *Xho*I and *Not*I. The *Xho*I and *Not*I fragments were then inserted into the corresponding sites of pCI‐neo to construct the hLF‐HSA expression vector pCI‐neohLF‐HSA.

### Protein overexpression

Plasmid vectors were introduced into PC‐14 cells by transfection with polyethylenimine Max (Polysciences Inc, Warrington, PA, USA) as described previously [24]. Briefly, PC‐14 cells were seeded at a density of 4 × 10^5^ cells/well in six‐well plates and incubated overnight. Expression vectors for mock (pCI‐neo only), hLF, HSA, or hLF‐HSA (4 μg each) were mixed with 10 μL of polyethylenimine Max in 500 μL serum‐free RPMI‐1640 medium and incubated for 20 min at 23 °C. The mixtures were added to the cells and incubated for 6 h at 37 °C. Next, the medium was replaced with fresh RPMI‐1640 containing 10% FBS and the cells were incubated for 48 h at 37 °C to allow expression. The cells were then lysed and subjected to western blotting.

### Okadaic acid treatment

PC‐14 cells were seeded in six‐well plates and incubated at 37 °C overnight. The cells were pretreated using a caveolae‐dependent endocytosis activator (1.25 μm okadaic acid) in RPMI‐1640 medium for 30 min. Subsequently, cells were incubated with 1.25 μm okadaic acid again in fresh RPMI‐1640 at 37 °C for an additional 4 h. The cells were then lysed and subjected to western blotting.

When the cells were treated with both hLF and okadaic acid, they were first pretreated with 1.25 μm okadaic acid for 30 min. Thereafter, 10 μm hLF was added along with the 1.25 μm okadaic acid at 37 °C for 4 h. When hLF‐HSA‐transfected cells were treated with okadaic acid, they were first incubated for 48‐h post‐transfection, then treated with 1.25 μm okadaic acid at 37 °C for 4 h.

### Statistical analysis

All data are expressed as the mean ± standard deviation (SD). Statistical differences were analyzed by Dunnett's test or unpaired Student's *t*‐test, as indicated in the figure legends. Statistical comparisons between groups were performed using a two‐way analysis of variance (ANOVA), followed by Tukey's test. Statistical analyses were performed using online statistical EZR software [25] (see https://www.jichi.ac.jp/saitama‐sct/SaitamaHP.files/statmedEN.html, Saitama Medical Center, Jichi Medical University, Saitama, Japan). Statistical significance was set at *P* < 0.05 (*P < 0.05, **P < 0.01, and ***P < 0.001).

## Results

### MMP1 is a key molecule in regulating PC‐14 cell migration

**Fig. 1:**
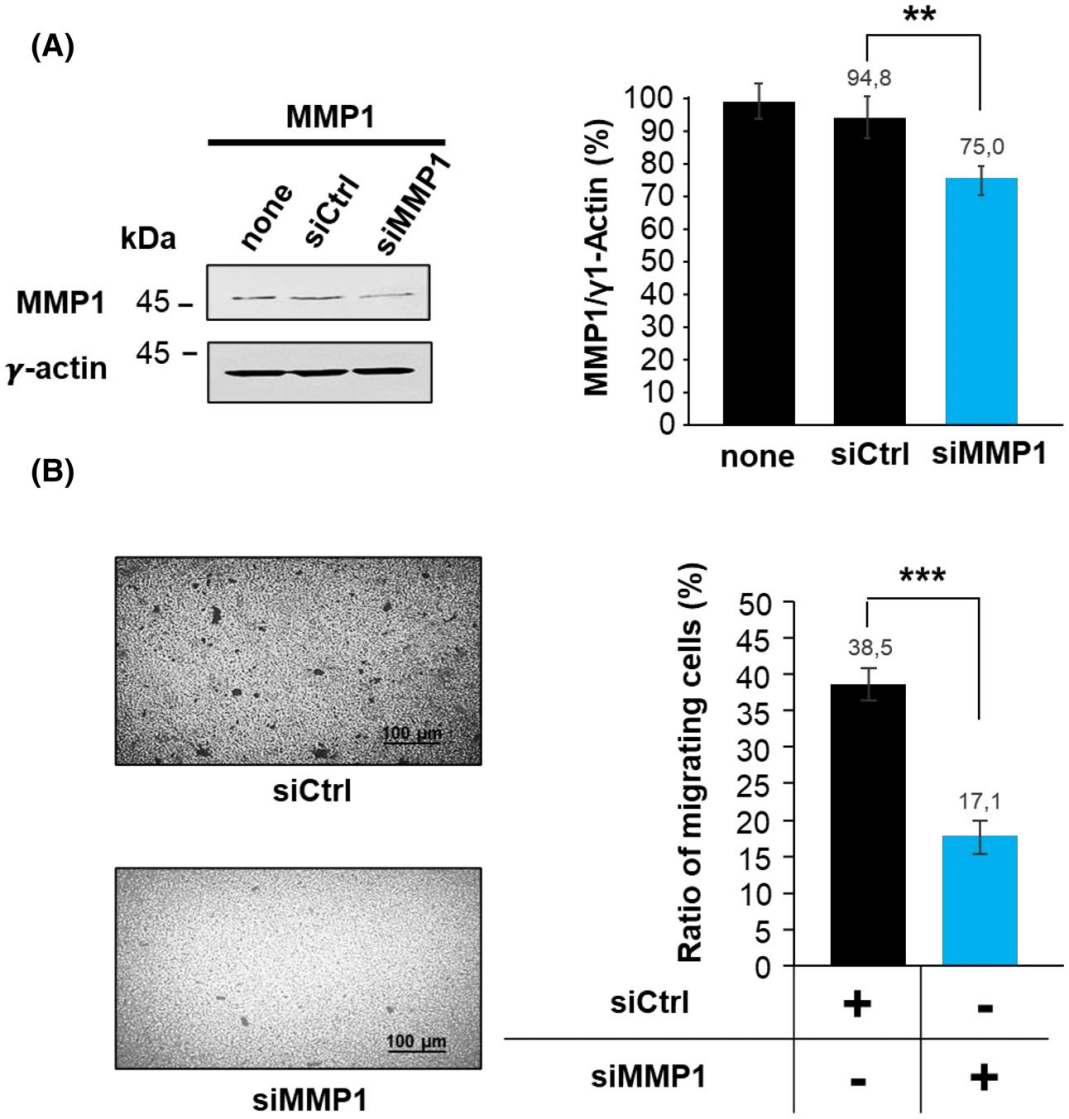
MMP1 is a key molecule in regulating PC‐14 cell migration. (A) MMP1 expression in PC‐14 cells following 48 h of treatment with siRNA. **P < 0.01 vs siCtrl (Student's *t*‐test). (B) PC‐14 cell migration was abolished by MMP1 silencing, as confirmed through the Boyden chamber assay. Scale bar, 100 μm. Data are presented as mean ± SD, with *n* = 3. ***P < 0.001 (Student's *t*‐test).

We have previously reported that MMP1 plays a role in PC‐14 cell migration, as indicated by its inhibition [21]. To confirm this, we used MMP1 siRNA (siMMP1)‐transfected PC‐14 cells (Fig. 1A) in the Boyden chamber assay. A significant reduction in cell migration was observed in MMP1‐silenced cells (Fig. 1B). Thus, MMP1 plays a pivotal role in the antimigratory activity of hLF‐HSA on PC‐14 cells. This indicates that MMP1 expression serves as a surrogate marker of PC‐14 cell migration.

### NHE 7 upregulation induced by hLF‐HSA leads to organelle alkalization in PC‐14 cells

**Fig. 2:**
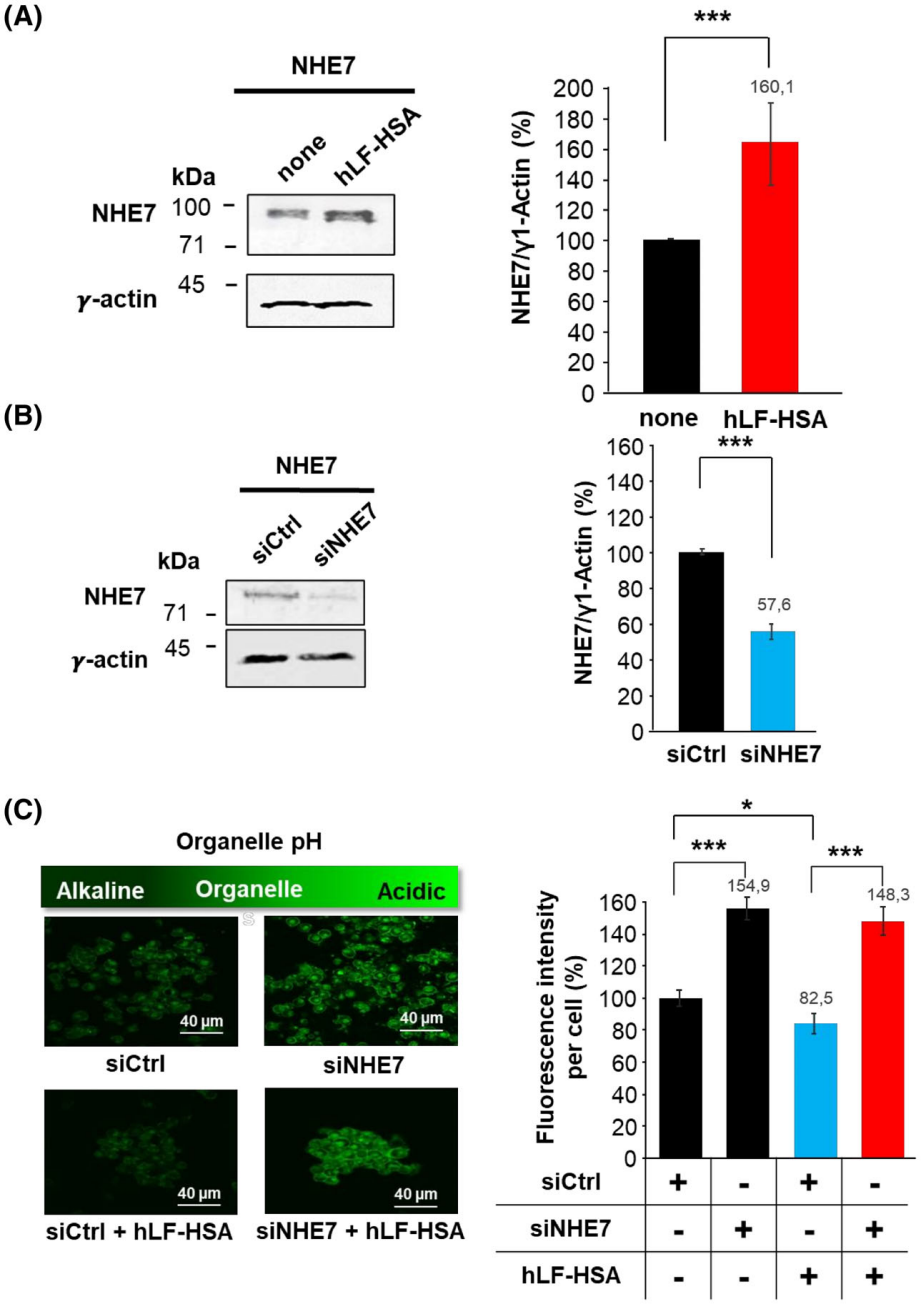
NHE7 upregulation induced by hLF‐HSA leads to organelle alkalization in PC‐14 cells. (A) hLF‐HSA treatment for 4 h revealed NHE7 upregulation. ***P < 0.001 (none vs hLF‐HSA [Student's *t*‐test]). (B) NHE7 expression in PC‐14 cells following 48 h of siRNA treatment. ***P < 0.001 (siCtrl vs siNHE7 [Student's *t*‐test]). (C) 4 hours of incubation with hLF‐HSA showed NHE7‐mediated organelle alkalization in PC‐14 cells. Results are presented as the mean ± SD (*n* = 3). Statistical significance was determined using two‐way ANOVA followed by Tukey's multiple comparisons test (*P < 0.05, ***P < 0.001).

In a recent study, we reported that organelle alkalization by hLF‐HSA led to organelle dysfunction, thereby inhibiting PC‐9 cell growth [10]. Thus, we focused on potential organelle alkalization induced by hLF‐HSA in PC‐14 cells. hLF‐HSA upregulated NHE7 expression in PC‐14 cells within 4 h (Fig. 2A), leading to organelle alkalization (lower panel of Fig. 2C).

Organelle alkalization induced by hLF‐HSA was abolished by the siRNA‐mediated knockdown of NHE7 (Fig. 2B,C). Therefore, hLF‐HSA induces organelle alkalization through NHE7 upregulation in PC‐14 cells within 4 h, indicating organelle dysfunction.

### NHE7 regulates the hLF‐HSA‐induced suppression of PC‐14 cell migration through MMP1 downregulation

**Fig. 3:**
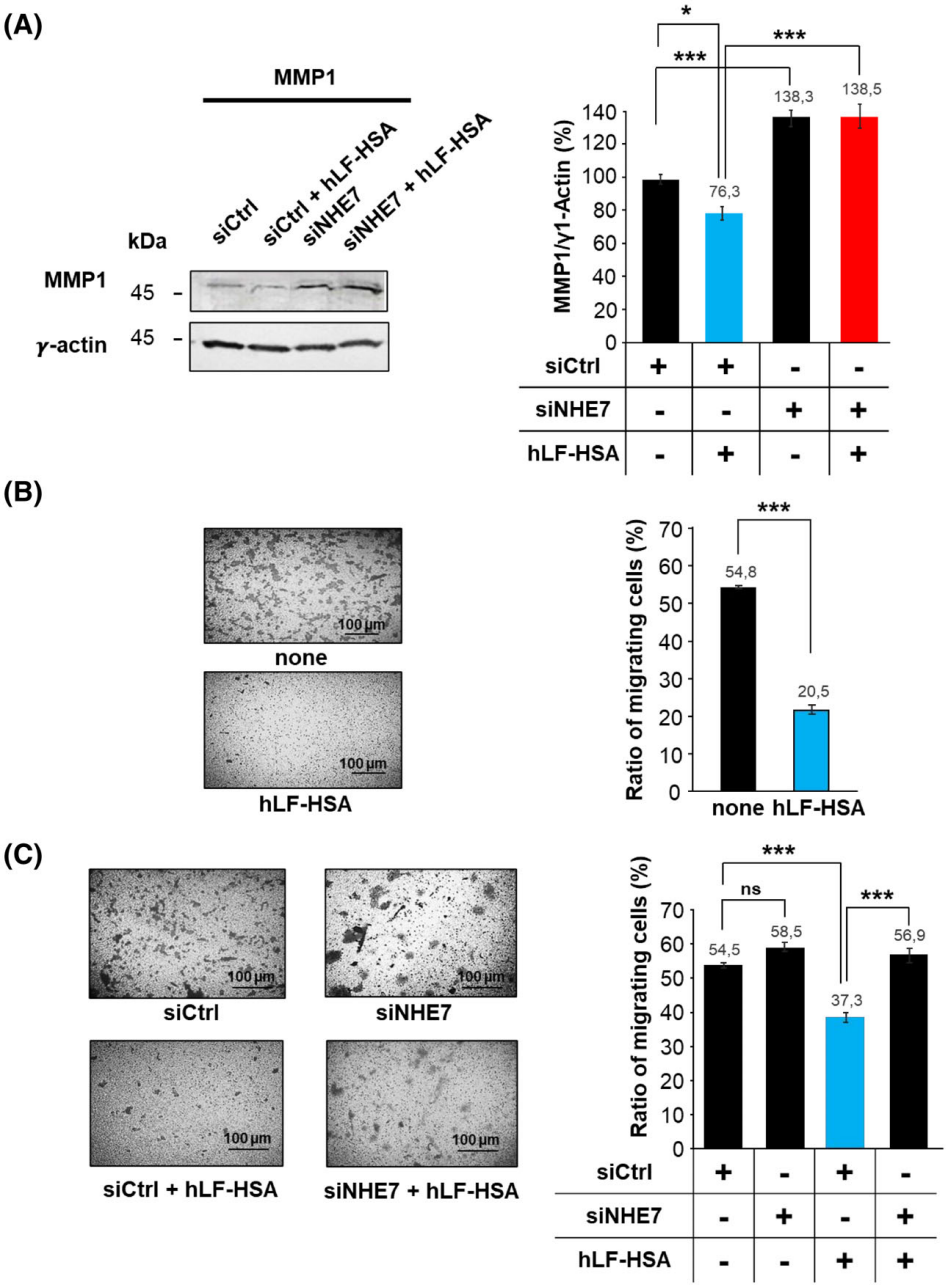
NHE7 regulates the hLF‐HSA‐induced suppression of PC‐14 cell migration through MMP1 downregulation. (A) siRNA‐mediated knockdown of NHE7 inhibited the downregulation of MMP1 expression by hLF‐HSA after 48 h. Statistical significance was determined using two‐way ANOVA followed by Tukey's multiple comparisons test (*P < 0.05, ***P < 0.001). (B) Antimigratory effects of hLF‐HSA on PC‐14 cells. ***P < 0.001 (none vs hLF‐HSA [Student's *t*‐test]) (C) Antimigratory effects of hLF‐HSA on PC‐14 cells were suppressed by siRNA knockdown of NHE7. Data are presented as mean ± SD (*n* = 3). Statistical significance was determined using two‐way ANOVA followed by unpaired Student's *t*‐test (nonsignificant (ns), ***P < 0.001).

Next, we investigated whether NHE7 modulates the expression of MMP1, a key player in PC‐14 cell migration, as an upstream signaling regulator. The hLF‐HSA‐induced MMP1 downregulation was suppressed by the siRNA‐mediated depletion of NHE7 (siNHE7) (Fig. 3A). Simultaneously, the suppression of PC‐14 cell migration by hLF‐HSA, which is a consequence of MMP1 downregulation, was inhibited by siNHE7 (Fig. 3C). When the cells were treated with hLF‐HSA, NHE7 was upregulated (Fig. 2A), whereas MMP1 was downregulated (Fig. 3A). Conversely, NHE7 downregulation by siRNA silencing resulted in MMP1 upregulation, even in the presence of hLF‐HSA (Fig. 3A). Boyden chamber assay showed a consistent result that hLF‐HSA suppressed cell migration in siNHE7‐transfected cells (Fig. 3C). Therefore, NHE7 negatively regulates MMP1 expression, probably through its role in organelle function in PC‐14 cells.

### Albumin fusion to hLF triggers the suppression of epithelial‐mesenchymal transition (EMT) in PC‐14 cells

**Fig. 4:**
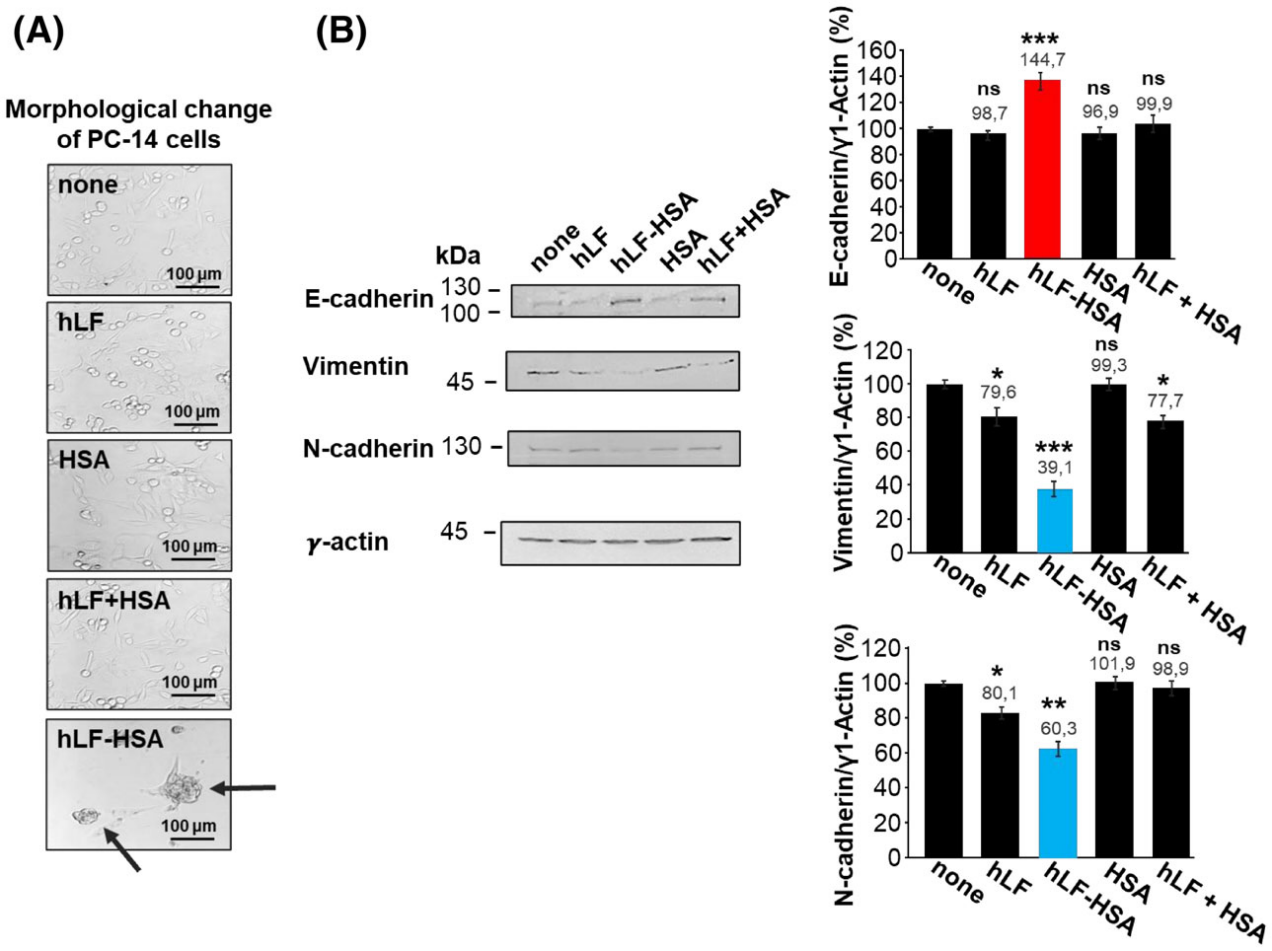
Albumin fusion to hLF inhibits PC‐14 cell migration through EMT suppression. (A) hLF‐HSA elicits a morphological change of PC‐14 cells into large, rounded clusters (arrow), symbolizing the reverse EMT process. Scale bar, 100 μm. (B) PC‐14 cells treated with hLF‐HSA revealed a significant increase in E‐cadherin expression and a corresponding decrease in vimentin and N‐cadherin expression, indicating a reverse EMT process (left). Relative band intensities are graphically represented (right). Data are presented as the mean ± SD (*n* = 3); nonsignificant (ns), *P < 0.05, **P < 0.01, and ***P < 0.001, vs none (Dunnett's test).

We observed morphological changes in the cells treated with hLF‐HSA. When the cells were exposed to hLF‐HSA, the cell shape changed from cobblestone (as seen in the untreated control, hLF, HSA, or the concurrent addition of hLF and HSA) to a colony‐forming morphology (Fig. 4A), indicating EMT reversal [26].

To confirm the possible reversal of EMT induced by hLF‐HSA, the expression of EMT biomarkers such as E‐cadherin, vimentin, and N‐cadherin [27] was examined. Compared with the untreated control, hLF‐HSA significantly enhanced E‐cadherin expression and reduced vimentin and N‐cadherin expression (Fig. 4B), indicating the induction of EMT reversal in PC‐14 cells. The hLF‐HSA‐induced EMT reversal represents an alternative anti‐migratory effect distinct from the direct reduction in MMP1 expression observed in PC‐14 cells.

### Silencing MMP1 elicited a reversal of the EMT process in PC‐14 cells

**Fig. 5:**
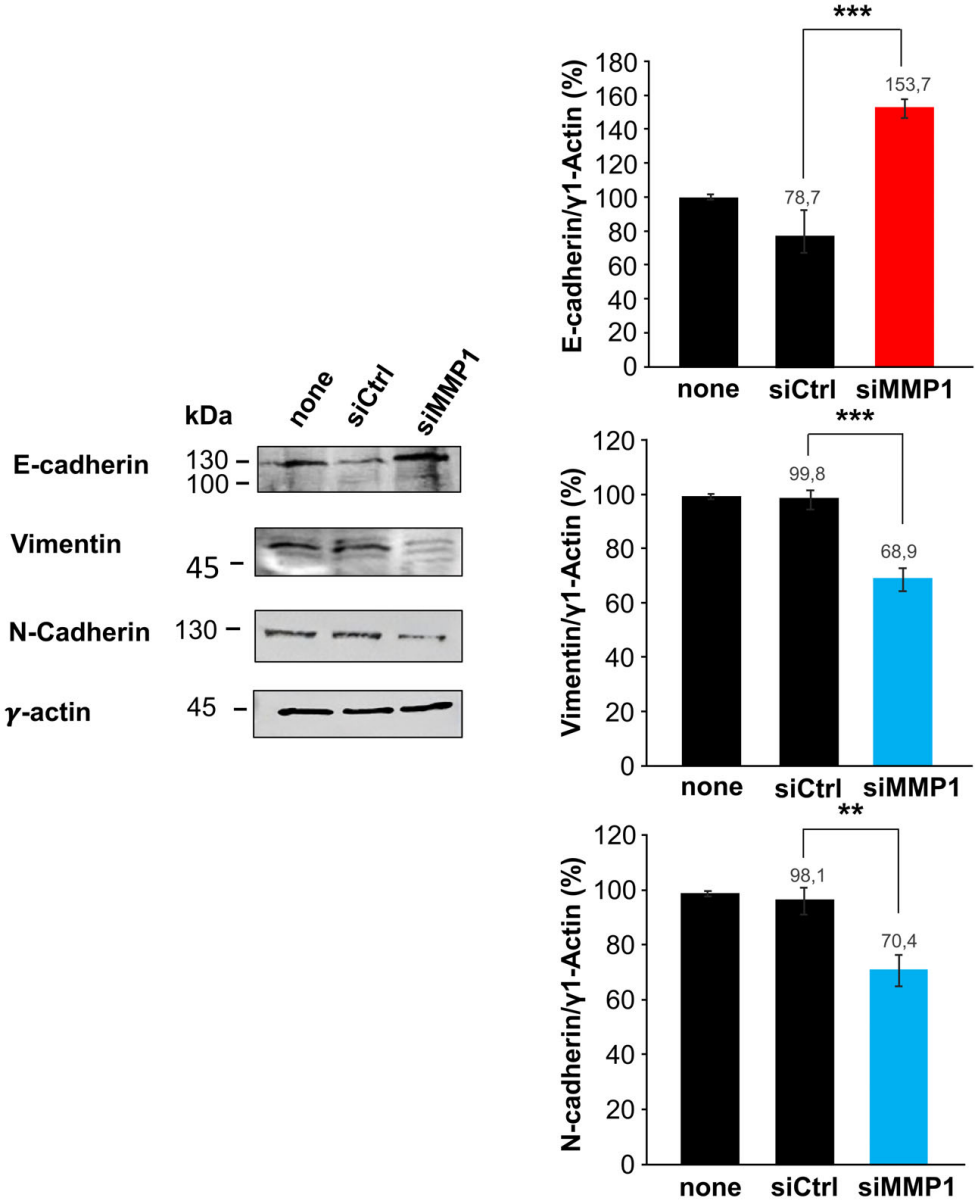
Silencing of MMP1 induced a reverse EMT process in PC‐14 cells, as indicated by changes in the expression of molecular biomarkers. Silencing of MMP1 induced a reverse EMT process in PC‐14 cells, as indicated by changes in the expression of molecular biomarkers. The relative band intensities are graphically represented (below). Data are presented as the mean ± SD (*n* = 3); **P < 0.01, ***P < 0.001, vs siCtrl (Student's *t*‐test).

The depletion of MMP expression has been reported to trigger the reversal of EMT in some cancer cell lines [28, 29, 30]. To determine whether hLF‐HSA‐induced MMP1 downregulation leads to EMT reversal in PC‐14 cells, we examined EMT biomarker expression in siMMP1‐transfected PC‐14 cells. The reversal of EMT was strongly observed in siMMP1‐treated cells, as confirmed using western blotting, which showed increased E‐cadherin expression and decreased vimentin and N‐cadherin expressions (Fig. 5). Therefore, hLF‐HSA‐induced depletion of MMP1 triggered the reversal of EMT.

### Caveolae‐mediated cellular uptake of hLF‐HSA is crucial for its antimigratory effects in PC‐14 cells

**Fig. 6:**
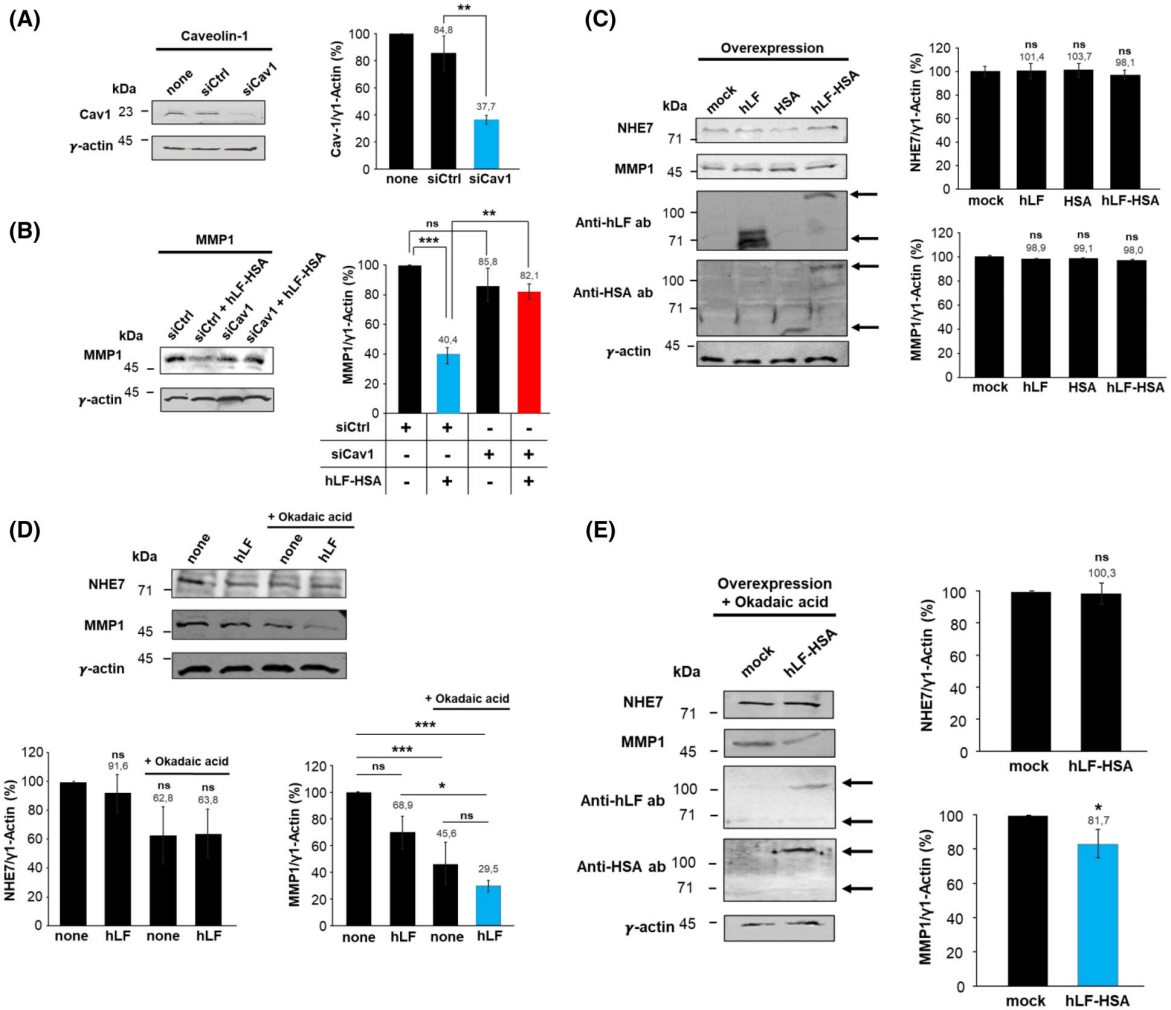
Caveolae‐mediated cellular uptake of hLF‐HSA is crucial for its antimigratory effects in PC‐14 cells. (A) Caveolin‐1 expression in PC‐14 cells following 48 h of siRNA treatment. **P < 0.01 vs siCtrl (Student's *t*‐test). (B) hLF‐HSA‐induced MMP1 downregulation was attenuated by siRNA knockdown of caveolin‐1. Statistical significance was determined using two‐way ANOVA followed by unpaired Student's *t*‐test (nonsignificant (ns), **P < 0.01, ***P < 0.001). (C) Overexpression of hLF‐HSA failed to upregulate NHE7 or downregulate MMP1 (ns vs mock, Dunnett's test). (D) Enhancement of CavME signaling by okadaic acid did not upregulate NHE7 (left) but significantly downregulated MMP1 (right), suggesting that CavME signaling by hLF‐HSA may contribute to its antimigratory effects in an NHE7‐independent fashion. Left, non‐significant (ns) vs none (Dunnett's test). Right, nonsignificant (ns), *P < 0.05, and ***P < 0.001 (Tukey's test). (E) hLF‐HSA overexpression and CavME signaling enhancement by okadaic acid did not upregulate NHE7 but downregulated MMP1 expression. Results are shown as the mean ± SD (*n* = 3). nonsignificant (ns), *P < 0.05 (mock vs hLF‐HSA [Student's *t*‐test]).

A previous study reported that hLF‐HSA is primarily taken up by cells through caveolae‐mediated endocytosis (CavME) [19]. Therefore, we investigated whether CavME is involved in the antimigratory effects of hLF‐HSA by silencing caveolin‐1 (siCav1; Fig. 6A), a key molecule in CavME [31]. The expression level of MMP1 was measured to assess its effect on the migration of PC‐14 cells. The hLF‐HSA‐induced MMP1 downregulation was blocked by siCav1 (Fig. 6B). Therefore, the antimigratory effect of hLF‐HSA on PC‐14 cells is dependent on CavME.

CavME involves two primary events: the intracellular presence of hLF‐HSA and/or its signaling. To determine which event contributes to the antimigratory activity induced by hLF‐HSA, we overexpressed hLF‐HSA to mimic its intracellular presence and/or used okadaic acid, a CavME activator, to stimulate CavME signaling [32]. Notably, hLF‐HSA overexpression, as well as hLF or HSA expression alone, did not significantly affect NHE7 or MMP1 expression (Fig. 6C). Okadaic acid treatment of PC‐14 cells did not result in NHE7 upregulation (Fig. 6D, left), unlike the hLF‐HSA treatment (Fig. 2A). This result did not change with the addition of 10 μm hLF (Fig. 6D, left). In contrast, treatment with okadaic acid led to MMP1 downregulation (Fig. 6D, right). MMP1 downregulation by okadaic acid was further enhanced with the addition of 10 μm hLF (Fig. 6D, right).

In contrast to hLF‐HSA treatment (Fig. 2A), the promotion of CavME signaling by okadaic acid did not result in NHE7 upregulation (Fig. 6D, left). Next, we evaluated the effects of both hLF‐HSA overexpression and enhancement of CavME signaling by okadaic acid. Although this approach led to MMP1 downregulation (Fig. 6E, lower right), it did not result in NHE7 upregulation (Fig. 6E, upper right).

Collectively, unlike the effects observed with hLF‐HSA treatment, the enhancement of CavME signaling by okadaic acid (Fig. 6D), as well as the combined effects of hLF‐HSA overexpression and CavME signaling enhancement (Fig. 6E), were insufficient to facilitate NHE7 expression. This indicates that factors other than CavME signaling and hLF‐HSA overexpression may be required. In addition, CavME signaling downregulated MMP1 expression in an NHE7‐independent manner (Fig. 6D), which may have contributed to the reduced migration of PC‐14 cells.

### Schematic diagram of the proposed mechanism underlying the antimigratory action of hLF‐HSA in PC‐14 cells

**Fig. 7:**
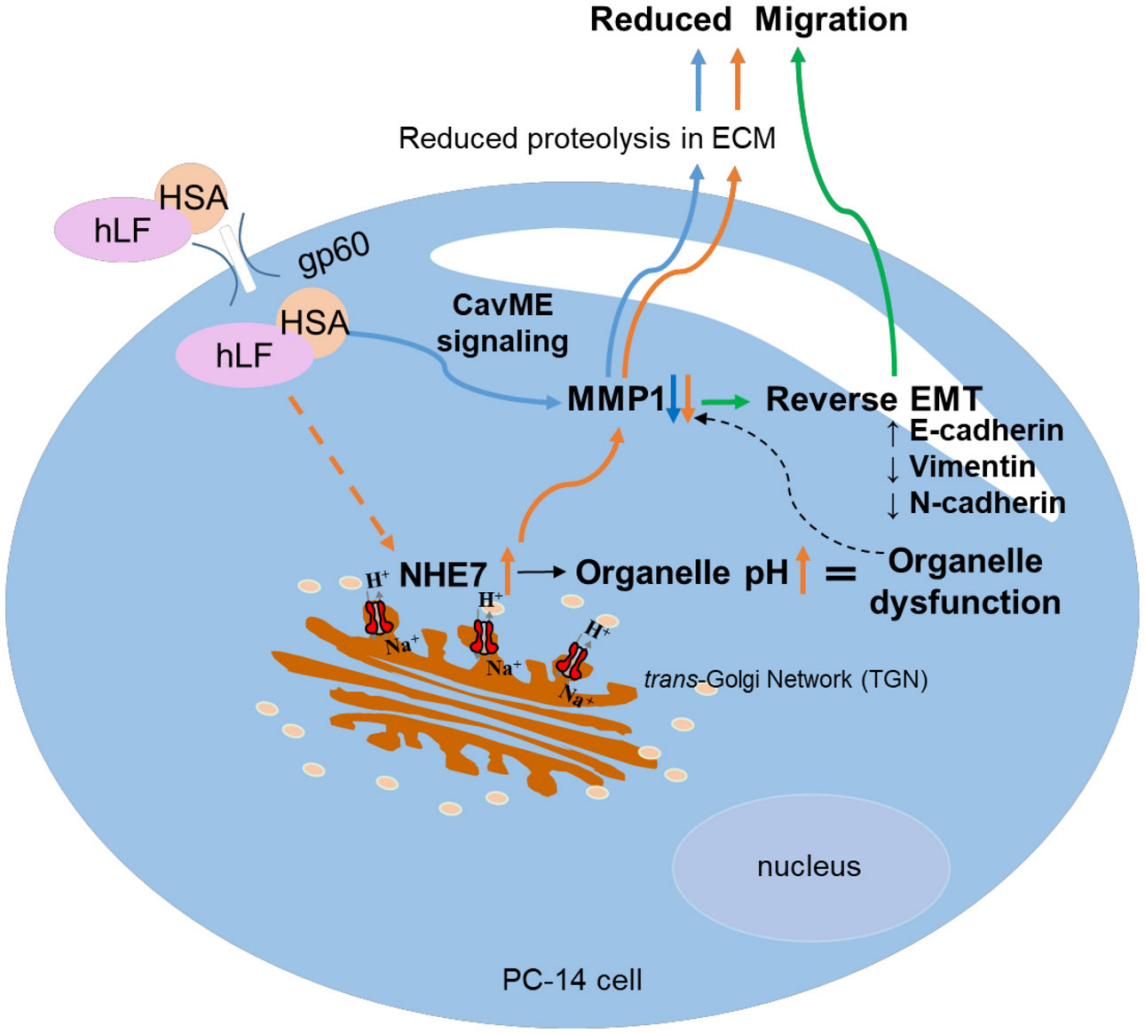
Schematic diagram of the proposed mechanism underlying the antimigratory action of hLF‐HSA in PC‐14 cells. Internalization of hLF‐HSA through the CavME‐dependent pathway enhances NHE7 expression, causing an increase in organelle pH, most likely within the TGN. pH increase impairs Golgi apparatus function, disrupting the Golgi‐associated secretome and resulting in the downregulation of MMP1. Reduced MMP1 secretion decreases proteolytic activity in the ECM, thereby inhibiting cancer cell migration (orange line). In addition, MMP1 suppression contributes to the reversal of EMT, thereby leading to a further reduction in PC‐14 cell migration (green line). Furthermore, CavME signaling triggered by hLF‐HSA independently reduces MMP1 levels in addition to NHE7 involvement, which may also contribute to the inhibition of cell migration (blue line).

Fig. 7 shows the proposed mechanism underlying the antimigratory effects of hLF‐HSA on PC‐14 cells. Intracellular introduction of hLF‐HSA through a CavME‐dependent pathway upregulates NHE7, causing an increase in organelle pH, likely in the TGN. Alkalization‐induced dysfunction of the Golgi apparatus disrupts the Golgi‐driven secretome, leading to MMP1 downregulation. Reduced MMP1 secretion decreases the proteolytic activity in the ECM, thereby suppressing cancer cell migration (orange line). MMP1 downregulation triggered EMT reversal, further reducing PC‐14 cell migration (green line). In addition, CavME signaling induced by hLF‐HSA led to MMP1 downregulation through an NHE7‐independent mechanism, which may also have contributed to the suppression of cell migration (blue line).

## Discussion

LF has been studied for its role in preventing metastasis and inhibiting cancer cell migration, as reported in several studies [17, 18]. However, our previous study showed that hLF promotes the migration of PC‐14 cells [21]. In contrast, hLF‐HSA suppressed cell migration by downregulating MMP1 expression, as confirmed using an MMP inhibitor [21]. In the present study, a significant decrease in cell migration was observed in the siMMP1‐transfected cells (Fig. 1B), strongly supporting the notion that MMP1 plays a key role in PC‐14 cell migration, consistent with previous studies [28, 33]. MMP1 is a collagenase that promotes cell migration by breaking down type I collagen, which creates pathways in the ECM that allow cancer cells to move through tissues [34]. Since hLF‐HSA is a potential Golgi‐targeting protein, it also exerted antiproliferative effects on cancer cells, reducing cell proliferation by approximately 32% under the same experimental conditions and time point (48 h) as those used in the Boyden chamber assay (Fig. S1). In contrast, hLF‐HSA treatment decreased the migrated area in the Boyden chamber assay by approximately 60% compared with control (Fig. 3B). Because the extent of migration inhibition substantially exceeded the reduction in total cell number, these data indicate that hLF‐HSA suppresses cell migration to a greater degree than can be explained by growth inhibition alone. This conclusion is consistent with our previous wound‐healing assay performed in the presence of mitomycin C, an inhibitor of cell proliferation, which demonstrated migration suppression under proliferation‐blocking conditions [21].

The EMT in cancer cells contributes to malignancy by enhancing cell migration and invasion [35]. Bovine LF suppresses EMT in cell carcinoma [36]. Treatment with hLF‐HSA reversed EMT in PC‐14 cells, as confirmed by relevant biomarker expressions (Fig. 4). In some cancer cells, MMP depletion triggers EMT reversal [30, 37]. In the present study, we verified that MMP1 silencing through siRNA treatment suppressed EMT in PC‐14 cells (Fig. 5). Therefore, hLF‐HSA‐induced MMP1 downregulation led to EMT reversal, revealing an additional antimigratory effect in PC‐14 cells.

However, the key question remains as to which factors regulate MMP1 expression in hLF‐HSA‐treated cells. The Golgi apparatus is widely recognized for its essential roles in oriented cell migration [38], and the secretion of proteins that help form the ECM [39]. A recent study reported Golgi‐mediated MMP1 secretion through a protein kinase D‐dependent secretome in triple‐negative breast cancer cell lines [40]. Therefore, we focused on the NHE7‐driven Golgi function in PC‐14 cells, particularly its role in regulating organelle pH and its potential impact on cell migration. Similar to PC‐9 cells [10], PC‐14 cells exhibited NHE7‐mediated organelle alkalization (Fig. 2). Disruption of organelle pH homeostasis due to hLF‐HSA‐induced alkalization may impair Golgi function and thereby influence the cancer cell secretome, potentially leading to downregulation of MMP1. Intracellular organelles, including the Golgi apparatus, are normally maintained at a mildly acidic pH, which is essential for their proper function [41]. Experimental alkalization of acidic organelles by weak bases such as NH_4_Cl and chloroquine induces Golgi dysfunction by impairing glycosylation, vesicle‐mediated transport, and Golgi‐associated proteolytic processing [42, 43, 44]. In this context, NHE7‐mediated organelle alkalization observed in the present study may similarly compromise Golgi function and affect Golgi‐dependent regulation of MMP1. The Golgi‐resident protein IMPAD1 has recently been reported to regulate lung cancer cell migration and invasion through Golgi‐mediated secretion of MMPs, including MMP1. Treatment with Brefeldin A, a well‐established Golgi‐disrupting agent, suppresses IMPAD1‐dependent MMP1 secretion and attenuates MMP‐driven invasion in lung cancer cells [45], supporting the concept that intact Golgi function is required for MMP1‐dependent migratory activity. Taken together, organelle dysfunction resulting from alkalization may contribute, at least in part, to the antimigratory effects of hLF‐HSA in PC‐14 cells.

Pharmacological targeting of the Golgi apparatus is challenging due to its essential functions in normal cells [5]. Dysregulation induced by hLF‐HSA in cancer cells, leading to an abnormally alkaline organelle environment, can potentially affect cellular processes in noncancerous cells. However, hLF‐HSA showed selective growth suppression in cancer cells compared to normal cells [20], strongly supporting its potential as a Golgi‐targeting anticancer biopharmaceutical.

Our findings, showing the antiproliferative [10, 19] and antimigratory (this study) effects of hLF‐HSA‐induced NHE7 upregulation in lung cancer cells, are in contrast with previous reports in which NHE7 was frequently and persistently upregulated in tumors, where it has been associated with malignant progression [9, 14, 15, 16]. Experimental NHE7 overexpression increased the invasive behavior of the breast cancer cell line MDA‐MB‐231 [12]. In contrast, our study focused on the transient NHE7 upregulation followed by a rapid pH shift induced by exogenous hLF‐HSA within 4 h (Fig. 2C). This rapid change, which disrupts organelle function, may suppress cancer cell migration rather than enhance malignancy.

The organelle pH regulated by hLF‐HSA may vary depending on the cancer type. In the pancreatic cancer cell line MIA PaCa‐2, hLF‐HSA induces organelle acidification, in contrast to the alkalization observed in PC‐14 cells [10]. This context‐dependent difference raises the question of whether hLF‐HSA‐induced organelle acidification exerts comparable antimigratory effects in MIA PaCa‐2 cells. However, our findings, together with evidence from pharmacological and genetic inhibition of NHE7 [9, 14, 15, 16], indicate that targeting organelle disruption may represent a potential antitumor strategy.

The CavME of hLF‐HSA is crucial for both its entry into PC‐14 cells [19] and its antimigratory effects (Fig. 6), as confirmed through caveolin‐1 knockdown. The caveolae‐dependent antimigratory effects of hLF‐HSA can be attributed to its intracellular presence and/or CavME signaling. However, hLF‐HSA overexpression (Fig. 6C,E) and/or CavME signaling stimulation by okadaic acid (Fig. 6D,E) did not result in elevated expression of NHE7, as observed with hLF‐HSA treatment. Therefore, additional signaling may be required to suppress PC‐14 cell migration.

Activation of CavME signaling by okadaic acid did not lead to NHE7 upregulation; however, it resulted in MMP1 downregulation (Fig. 6D). This finding indicates that MMP1 downregulation mediated by hLF‐HSA‐induced CavME signaling may contribute to the antimigratory effects of hLF‐HSA on PC‐14 cells. The link between CavME signaling and MMP1 downregulation in PC‐14 cells is further supported by a previous study showing that caveolin‐1, a key structural component of caveolae, acts as a negative regulator of MMP1 in human dermal fibroblasts [46]. Together, NHE7‐dependent MMP1 downregulation and CavME signaling‐induced MMP1 suppression may play a role in the inhibition of cell migration in lung cancer cells.

This study was limited to the PC‐14 lung adenocarcinoma cell line for mechanistic investigations; therefore, the generalizability of these findings to other lung adenocarcinoma models or cancer types remains to be determined. Additionally, our conclusions are based solely on *in vitro* migration assays. While suppression of migration is suggestive of antimetastatic potential, it does not directly equate to inhibition of metastasis, which would require *in vivo* validation using orthotopic or xenograft models.

## Conclusion

In the present study, we elucidated the mechanism underlying the antimigratory effect of hLF‐HSA, which involves NHE7‐mediated organelle alkalization and CavME signaling in lung adenocarcinoma PC‐14 cells. Although further investigation is required to fully validate the range of antimigratory properties, our findings indicate that the disruption of organelle pH homeostasis induced by hLF‐HSA provides potential approaches to suppress cancer cell migration.

## Conflict of interest

Atsushi Sato is the founder of S&K Biopharma Inc (Kawasaki, Kanagawa, Japan). The authors disclose that the hLF‐HSA experimental sample used in this study is protected under Japanese Patent No. 7142915 and US Patent No. 11041014, which is held by S&K Biopharma Inc.

## Author contributions

HN: data curation, formal analysis, investigation, visualization, and writing of the original draft. MK: formal analysis, investigation, reviewing the manuscript, and editing. DK: investigation and visualization. AS: conceptualization, data curation, formal analysis, project administration, supervision, visualization, writing of the original draft, reviewing the manuscript, and editing.

## Supporting information


**Fig. S1.** Growth‐inhibitory effects of hLF and hLF‐HSA at 48 h. PC‐14 cells were seeded in 96‐well plates at a density of 1.5 × 10^4^ cells per well and incubated overnight. Subsequently, the culture medium was replaced with RPMI‐1640 medium supplemented with 10% FBS and 5 μm hLF or hLF‐HSA, and the cells were cultured at 37 °C in a 5% CO₂ incubator for 48 h. Cell proliferation was analyzed using the Cell Counting Kit‐8 (Dojindo Laboratories). Data are presented as the mean ± SD (*n* = 3); ns, not significant; **, *P* < 0.01 (vs none, Dunnett's test).

## Data Availability

The data supporting the findings of this study are available from the corresponding author upon reasonable request.
