# Supplementary material for: Suppression of lung adenocarcinoma migration through organelle alkalization by human lactoferrin – albumin fusion
Source: FEBS Open Bio. 2026 Mar 24:10.1002/2211-5463.70237. Online ahead of print. doi: 10.1002/2211-5463.70237 (PMC13399163; doi:10.1002/2211-5463.70237)
Supplement: Supplementary file 1 — Fig. S1. Growth‐inhibitory effects of hLF and hLF‐HSA at 48 h. PC‐14 cells were seeded in 96‐well plates at a density of 1.5 × 10^4^ cells per well and incubated overnight. Subsequently, the culture medium was replaced with RPMI‐1640 medium supplemented with 10% FBS and 5 μm hLF or hLF‐HSA, and the cells were cultured at 37 °C in a 5% CO₂ incubator for 48 h. Cell proliferation was analyzed using the Cell Counting Kit‐8 (Dojindo Laboratories). Data are presented as the mean ± SD (n = 3); ns, not significant; **, P < 0.01 (vs none, Dunnett's test). [file FEB4-9999-0-s001.pptx]

## Slide 1
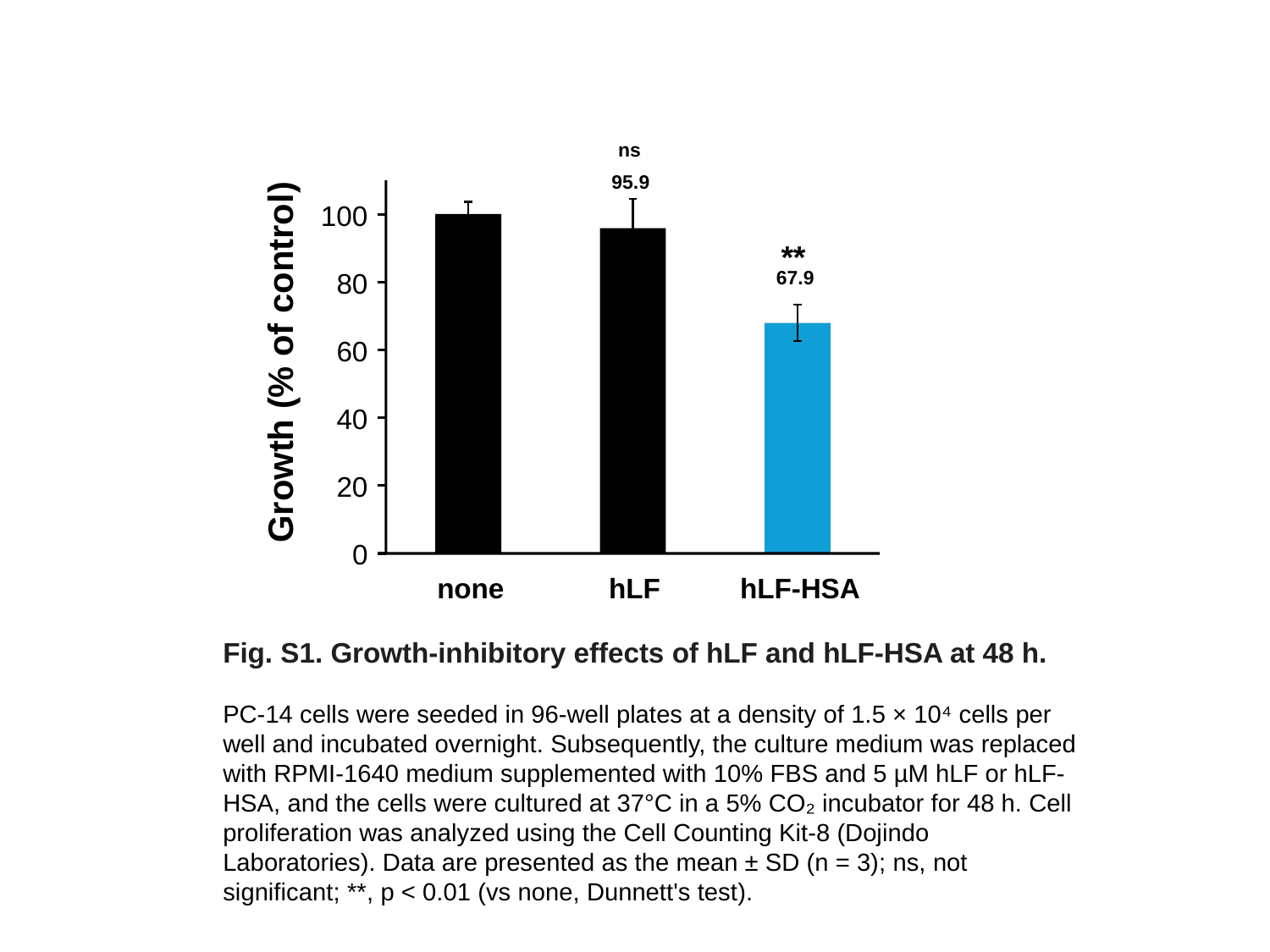

ns
95.9
**
67.9
Growth (% of control)
100
80
60
40
20
0
none
hLF
hLF-HSA
Fig. S1. Growth-inhibitory effects of hLF and hLF-HSA at 48 h.
PC-14 cells were seeded in 96-well plates at a density of 1.5 × 10⁴ cells per well and incubated overnight. Subsequently, the culture medium was replaced with RPMI-1640 medium supplemented with 10% FBS and 5 µM hLF or hLF-HSA, and the cells were cultured at 37°C in a 5% CO₂ incubator for 48 h. Cell proliferation was analyzed using the Cell Counting Kit-8 (Dojindo Laboratories). Data are presented as the mean ± SD (n = 3); ns, not significant; **, p < 0.01 (vs none, Dunnett's test).
